# Recruitment and metabolomics between *Canna indica* and rhizosphere bacteria under Cr stress

**DOI:** 10.3389/fmicb.2023.1187982

**Published:** 2023-08-15

**Authors:** Huan Mao, Wei Zhao, Xiuqin Yang, Luying Sheng, Sixi Zhu

**Affiliations:** The Karst Environmental Geological Hazard Prevention of Key Laboratory of State Ethnic Affairs Commission, College of Eco-Environment Engineering, Guizhou Minzu University, Guiyang, China

**Keywords:** chromium, *Canna indica*, rhizosphere, microbiome, metabolomics

## Abstract

It is of positive significance to explore the mechanism of antioxidant and metabolic response of *Canna indica* under Cr stress mediated by rhizosphere niche. However, the mechanisms of recruitment and interaction of rhizosphere microorganisms in plants still need to be fully understood. This study combined physiology, microbiology, and metabolomics, revealing the interaction between *C. indica* and rhizosphere microorganisms under Cr stress. The results showed that Cr stress increased the content of malondialdehyde (MDA) and oxygen-free radicals (ROS) in plants. At the same time, the activities of antioxidant enzymes (SOD, POD, and APX) and the contents of glutathione (GSH) and soluble sugar were increased. In addition, Cr stress decreased the α diversity index of *C. indica* rhizosphere bacterial community and changed its community structure. The dominant bacteria, namely, *Actinobacteriota, Proteobacteria*, and *Chloroflexi* accounted for 75.16% of the total sequence. At the same time, with the extension of stress time, the colonization amount of rhizosphere-dominant bacteria increased significantly, and the metabolites secreted by roots were associated with the formation characteristics of *Proteobacteria, Actinobacteria, Bacteroidetes*, and other specific bacteria. Five critical metabolic pathways were identified by metabolome analysis, involving 79 differentially expressed metabolites, which were divided into 15 categories, mainly including lipids, terpenoids, and flavonoids. In conclusion, this study revealed the recruitment and interaction response mechanism between *C. indica* and rhizosphere bacteria under Cr stress through multi-omics methods, providing the theoretical basis for the remediation of Cr-contaminated soil.

## Graphical Abstract

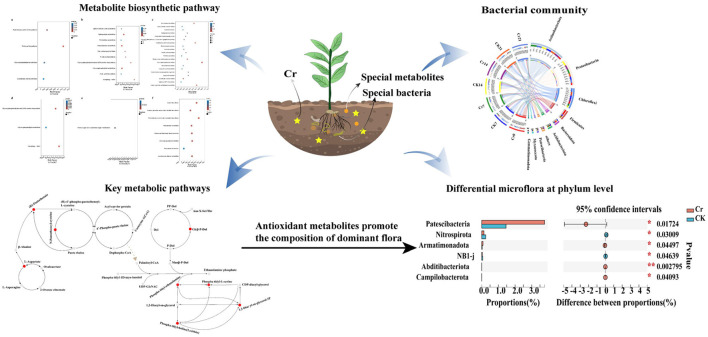

## 1. Introduction

With the rapid development of industry, heavy metal pollution in the environment is becoming more and more serious (Uchimiya et al., 2020). Heavy metals, such as lead (Pb), cadmium (Cd), chromium (Cr), and mercury (Hg), are characterized by high persistence, wide range, and strong toxicity (Yin et al., 2023). Among them, Cr has substantial bioavailability and mobility in the environment, which will not only cause harm to soil and plant health but also indirectly harm human health through enrichment (Morais et al., 2021). Cr usually exists in the oxidation state of Cr^3+^ and Cr^6+^, among which Cr6^+^ has more potent toxicity and migration ability (Husain et al., 2021). Cr stress will destroy the homeostasis of physiological activities such as photosynthesis, respiration, oxidative stress, and the enzymatic reaction of plants, failing the average growth of plants (Hu et al., 2020). At the same time, it can also damage plant genetic information and cause plant mutations (Mumtaz et al., 2022). Therefore, studying Cr-contaminated soil bioremediation technology is the top priority.

At present, the remediation of soil Cr pollution mainly includes chemical conversion, biological conversion, and physical remediation (Prasad et al., 2021). Among them, chemical transformation and physical remediation could be more efficient in the remediation of Cr-contaminated soil, and secondary pollution and waste of resources will be generated during remediation (Muthusaravanan et al., 2018). However, microbial and plants used in bioremediation work well to solve these problems. Among them, *Bacillus megatherium PMW-03* (Guo et al., 2021), *Pannonibacter phragmitetus BB* (Liao et al., 2014), *Bacillus, Reibacillus*, and *Staphylococcus* (Pradhan et al., 2020) can significantly enrich and transform the heavy metal Cr to reduce its biotoxicity. At the same time, plants can use root cell walls, stems, leaves, and other organs to store Cr or form complexes and chelates with its antioxidant substances, thus reducing the toxic effect of Cr on plants (Chaâbane et al., 2020; Tripathi et al., 2023).

The rhizosphere is a microhabitat formed by the interaction between plant root exudates and soil microorganisms, and their mutualism changes the whole microenvironment (Wang et al., 2023). Plant physiological mechanisms control root exudates, and exudates induce microbial activity, which enables plants to cope better with stressed environments (Wu et al., 2023). Root exudates include amino acids, sugars, vitamins, fatty acids, and organic acids, while differentially expressed metabolites (flavonoids, citric acid, and camelina) are secreted to recruit specific microorganisms (Santoyo, 2022). In addition, plant species can specifically interact with specific microbiomes. For example, legumes form symbionts with rhizobia bacteria, whose secretions promote the growth of legumes' root hairs (Thornton and Nicol, 1936). At the same time, the types of plant root exudates were also affected by stress factors. For example, a recent review showed that drought enhances root secretion of abscisic acid, proline, and betaine. To cope with phosphorus deficiency, plants tend to increase the secretion of citric acid, malic acid, or oxalic acid to enrich rhizosphere organic carbon and attract beneficial microorganisms (Chai and Schachtman, 2022). Root exudates regulate the toxicity and migration of Cr by complexation or chelation. For example, in the study on treating Cr-contaminated soil by compost and phytoremediation, low-molecular-weight organic acids (citric acid, malic acid, tartaric acid, and oxalic acid) secreted by herbaceous plants increased the bioavailability of Cr and promoted the desorption and chelation process of Cr (Chen et al., 2018). Meanwhile, plant growth-promoting bacteria (PGPB) can promote plant growth under Cr stress because PGPB changes plant photosynthesis and antioxidant stress response (Vishnupradeep et al., 2022). Therefore, different microorganisms induced by plant root secretions to regulate plant homeostasis under Cr stress were studied to elucidate related detoxification mechanisms and rhizosphere microbial community changes.

*Canna indica* is a perennial herbaceous plant with a large leaf area, developed root system, and strong adaptability to the growing environment (Luo et al., 2022a,b). *C. indica* is effective in heavy metal adsorption and nitrogen removal in constructed wetlands and significantly contributes to heavy metal adsorption capacity and soil fertility after airbrushing mycorrhizal fungi (AMF) colonization (El Faiz et al., 2015; Ghezali et al., 2022). However, the effects and mechanisms of differentially expressed metabolites on rhizosphere-related microbial communities under heavy metal stress still need further study. Therefore, this study conducted a combined analysis of the microbiome and metabolome in the rhizosphere of *C. indica* under Cr stress in order to reveal the recruitment and interaction response mechanism between wetland plant *C. indica* and rhizosphere bacteria under Cr stress. Therefore, this study aimed to (1) determine the stress expression of *C. indica* in response to Cr stress; (2) analyze the changes in rhizosphere microbial community diversity and structure of *C. indica* under Cr stress, as well as the recruitment of dominant and plant-promoting bacteria by plant root metabolites, and reveal the mechanism of plant–microbial interaction; and (3) reveal the biosynthetic pathway of *C. indica* root metabolites by metabolomics analysis.

## 2. Materials and methods

### 2.1. Plant cultures

The *C. indica* seedlings (those with similar growth conditions and 10 cm tall) are purchased from Songnan Seed and Seedling Company of Luzhi Town, Suzhou City. To disinfect the surface of the *C. indica* seedlings purchased (75% ethanol for 10 s, 1% sodium hypochlorite solution for 15 min) (Hu et al., 2021), spray the disinfection liquid on the surface of the seedlings and then clean the surface with deionized water five times. The washed seedlings were sown on potting soil (a 500 g pot of soil) in the greenhouse. Greenhouse conditions are controlled at 12 h of light cycle, light intensity 176 μmol^*^m^2*^s^−1^, 25°C constant temperature. All seedlings were cultured for 30 days and subjected to a stress experiment with Cr addition. In addition, during the seedling culture period, Hoagland solution (15 ml) and water (15 ml) were added into the soil culture pot every 15 and 3 days successively. If Cr was added to the stress experiment, the Hoagland nutrient solution was not required (Si et al., 2022; Wei et al., 2023c).

### 2.2. Experimental design

A total of 21 *C. indica* seedlings with the same size and growth status were randomly divided into seven groups, with three plants in each group. Each group had three replicates. Three replicates are one pot and one seedling, a total of three pots. Four groups were the control group (0 mg^*^kg^−1^ K_2_Cr_2_O_7_), and three groups were the experimental group (100 mg^*^kg^−1^ K_2_Cr_2_O_7_). After acclimating *C. indica* seedlings, a Cr stress test was conducted. Samples were collected in the control and experimental groups on days 0, 7, 14, and 21, respectively. Plant leaves were cut with sterile scissors to collect plant leaves and facilitate the sequencing of subsequent biochemical indicators. Disassemble the potted plant and separate it from the soil as follows. First, most of the soil was removed from the potted plant with a sterile knife. Then, the plant is lifted and patted to remove the fixed soil on which the roots are attached, and the soil on which the roots are attached is collected as rhizosphere soil. Finally, 50 mesh sterile sieves and tweezers were used to screen the fibrous roots. After the rhizosphere soil is collected, plant roots are collected (Wei et al., 2023). The roots were cleaned five times with deionized water and immediately preserved in liquid nitrogen. At the same time, plant roots obtained from rhizosphere soil collection with a sterile sieve were also cleaned and put into root samples. The sampled rhizosphere soil, plant roots, and plant leaves were placed in clean sealed bags, immediately frozen with liquid nitrogen, and finally stored in a −80°C refrigerator. Samples were collected for microbiome and metabolomics analysis. After the rhizosphere soil samples were used for microbiome analysis, the remaining rhizosphere soil was used for physicochemical properties determination (Sixi et al., 2023).

### 2.3. Determination of chromium content

After collecting plant samples, collect potting soil samples. Then, place all soil samples on yellow paper in the vent to air dry. The soil was then air-dried, pounded, and screened with 100 mesh, and the sample was placed in a new sealed bag for subsequent measurements. An amount of 0.5 g of soil sample was weighed in the digestion tube, reverse king water (concentrated hydrochloric acid ratio of concentrated nitric acid is 1:3) was added in the digestion tube, and then the digestion tube was placed on the electric heating plate under the fume hood for heating digestion. After digestion at 160°C for 1 h, 3 ml perchloric acid was added, and the digestion was heated again at 200°C for 1 h. After digestion, the liquid was extracted in the test tube, and the Cr ion was fixed with 1% dilute nitric acid. After sampling the leaves of the plants, dry them directly in the oven (105°C) for 12 h. The blade diameter should be <0.02 mm when grinding the dried leaf sample. The mashed leaves (0.5 g each) were added with 37% HCl and 63% HNO_3_, sealed, and placed in the oven (105°C) for digestion for 10 h, and then, the suspension was diluted with 3 ml HNO_3_. Cr content was analyzed by inductively coupled plasma mass spectrometry (ICP-MS; Agilent,7800 ICP-MS, USA) (Karnaeva et al., 2021).

### 2.4. Physiological and biochemical indexes

#### 2.4.1. Physicochemical property

The pH and conductivity (EC) of soil samples were determined by the potentiometric method, while soil organic matter (SOM) was determined by K_2_Cr_2_O_7_-H_2_SO_4_ oxidation-external heating method (Bao, 2000).

#### 2.4.2. Photosynthetic pigment and soluble sugar content

*Measurement of chlorophyll content*: First, 0.1 g of *C. indica* leaves was weighed, the leaves were placed into the grinder, a small amount of powder (about 50 mg) and a small amount of chlorophyll determination buffer were added, and the leaves were ground into a homogenate. Then, the homogenate was placed in a 10 ml centrifuge tube. The chlorophyll determination buffer was added to the 10 ml centrifuge tube and then treated statically away from light (5 min−2 h). The pigment extraction is completed when the homogenate sample tissue is close to white. Finally, the supernatant liquid was taken after centrifugation and set at 665 and 649 nm, respectively, to determine the absorbance of chlorophyll a and chlorophyll b. The carotenoid content was determined in the same way as the chlorophyll content. However, the chlorophyll-assay buffer was changed to the carotenoid assay buffer at the grinding and resting stages (Meng et al., 2022). To accurately detect soluble sugar content in samples, the anthrone colorimetric method is used (Wei et al., 2023a). The specific scheme is to weigh a 0.1 g plant sample in a test tube and add distilled water until the test tube is full. It is then boiled at 100°C for 20 min in a 100 ml volumetric bottle and cooled at a constant volume. The process is carried out in boiling water after 5 ml of anthrone reagent is added to 1 ml of sample solution. After that, the absorbance of 620 nm was measured, and the soluble sugar content was calculated.

#### 2.4.3. Antioxidant enzyme content, GSH content, ROS, and MDA levels

The test kit for determining the content of superoxide dismutase (SOD), peroxidase (POD), and ascorbate peroxidase (APX) in *C. indica* plants was provided by Suzhou Kaming Biotechnology Co., LTD., (www.cominbio.com). Condition 5 (BDTS, USA) multifunctional ELISA for detecting absorbance is set at 560, 240, 470, and 290 nm. At the same time, the content of reduced glutathione (GSH), malondialdehyde (MDA), and reactive oxygen species (ROS) in plants is determined according to the kit. When the detection conditions were set, GSH was measured at 412 nm, while MDA was measured at 600 nm and 532 nm. In contrast, ROS levels were measured by a multifunctional ELISA (Berten, USA) for 10 min, with an excitation wavelength of 499 nm and an emission wavelength of 521 nm. The absorbance measured can be converted into the content according to the formula given by the kit (Wei et al., 2023b).

#### 2.5. DNA extraction and bacterial community analysis

Total genomic DNA from the *C. indica* rhizosphere soil samples was extracted using a FastDNA SPIN Kit for Soil (Qbiogene Inc., USA) following the manufacturer's instructions and sent to Majorbio Bio-pharm Technology, Shanghai, for sequencing. *Sequencing interval*: 338F and 806R, the primer's name was 338F/806R. The hypervariable region V3–V4 of the bacterial 16S rRNA gene was amplified with primer pairs 338F (5′-ACTCCTACGGGAGGCAGCAG-3′) and 806R (5′-GGACTACHVGGGTWTCTAAT-3′) by an ABI GeneAmp^®^ 9700 PCR thermocycler (ABI, CA, USA). Samples were randomly selected for pre-experiments to ensure that the majority of samples in the lowest number of cycles could be amplified to the appropriate concentration of products. After the preliminary experiment, TransGen ap 221-02 was used in the formal PCR test: TransStart Fastpfu DNA Polymerase, 20 μl reaction system (Supplementary Table S1). PCR instrument used: ABI GeneAmp^®^ 9700; PCR reaction parameters: a. 1 × (3 min at 95°C), b. cycle number × (95°C 30 s; Annealing 80°C 30 s; 45 s at 72°C), and c. 10 min at 72°C, 10°C until halted by the user. PCR products are detected by 2% agarose gel electrophoresis. The PCR products were quantified using the QuantiFluor™ ST Blue fluorescence quantification system (Promega) about the initial quantitative results of electrophoresis and then mixed preoperatively according to the sequencing volume requirements of each sample. Illumina libraries were then constructed according to kit instructions (TruSeqTM DNA Sample Prep Kit). Meanwhile, part of the analysis software is provided in Supplementary Table S2. The Major Cloud platform was used to conduct OTU cluster analysis of sequencing results (based on Research software), optimize sequences to extract non-repeating sequences, select sequences with more than 97% similarity with representative sequences, and calculate OTU number (i.e., the observed abundance sobs value). At the same time, all OTU numbers were drawn flat and then subjected to alpha diversity analysis to analyze species richness and diversity (Wang et al., 2021). At the same time, the obtained microbial information data have been uploaded to the NCBI database (PRJNA990318).

#### 2.6. Metabolome analysis

The collected plant root tissue samples were weighed and stored in liquid nitrogen freezing. Before extracting metabolites for analysis, root samples were air-dried and ground into powder. The metabolites were extracted by adding 0.6 ml methanol/water (7:3, v/v) and 0.3 mg/mL L^−2^-chlorophenylalanine solution to 60 mg of ground powder as indicated by the test. The ultrasound lasted for 30 min. After incubation at −20°C for 20 min, the extract was centrifuged at 4°C (14,000 rpm) for 10 min, and the supernatant with 200 μl centrifugation was filtered (0.2 μm). Filtrate detection was performed using the Waters VION IMS Q-TOF mass spectrometer (Waters Corporation, Milford, MA, USA) platform with an electric spray interface (Wu et al., 2023).

#### 2.7. Statistical analysis

Chen-Shapiro and Bartlett were used to test the normality and homogeneity of experimental data, and non-parametric tests were applied to the data that did not obey the normal distribution. All statistical analyses were performed using IBM SPSS Statistics 27.0 for the Windows software package. All data values were expressed as mean ± standard deviation, and a normality check was completed before the two-way ANOVA. In addition, *p* < 0.05 was the test of significance. Plotting was done using Windows Statistics Software Origin 2022 and Adobe Illustrator CC 2022. All microbial analyses and metabolome visualizations (Venn maps, heat maps, and bubble maps) are produced using an online platform (www.majorbio.com).

## 3. Results

### 3.1. Physiological changes and Cr accumulation in plants

**Figure 1:**
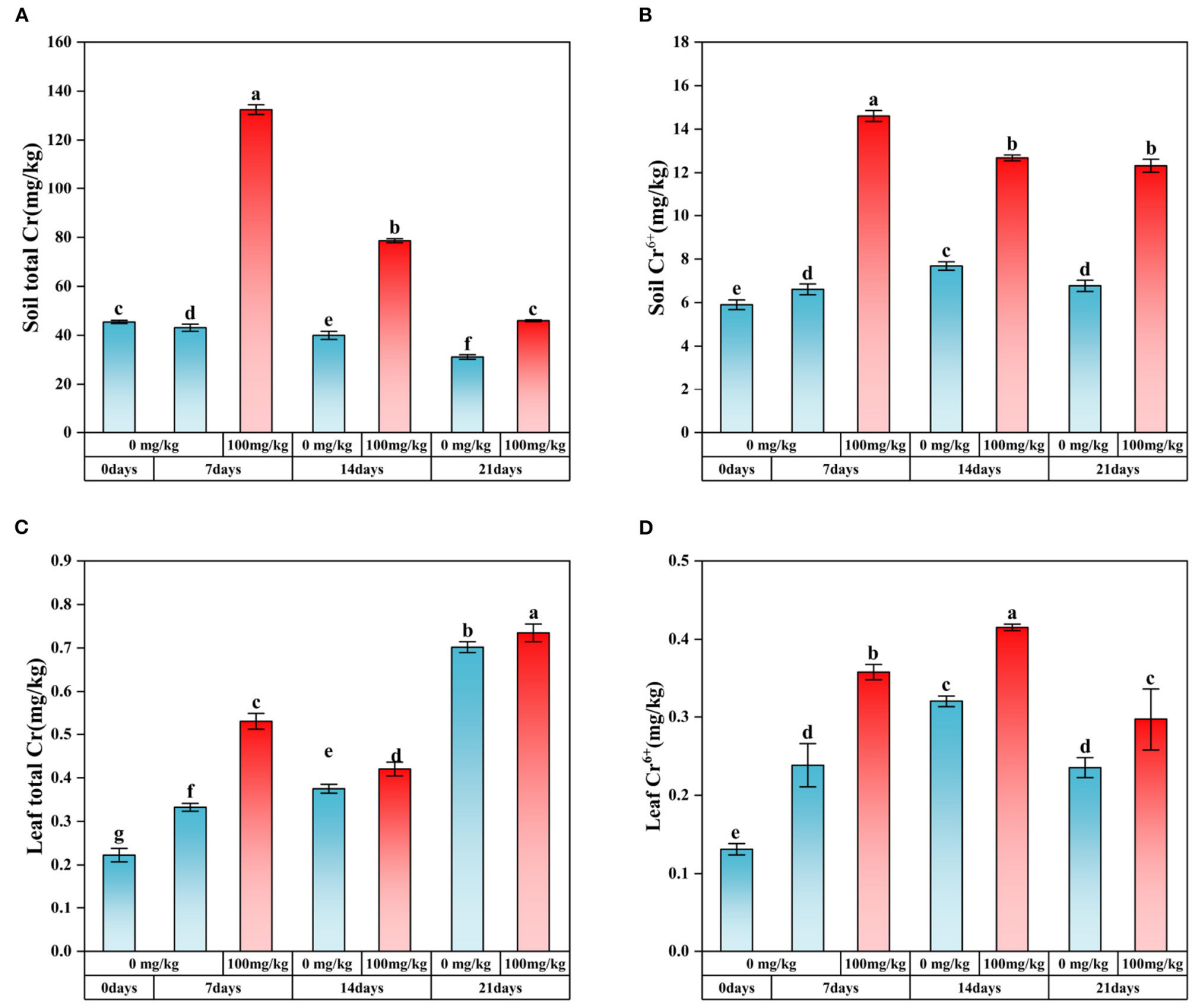
Content of Cr and Cr(VI) in soil and *C. indica* leaves. There was no significant difference in data within the same interval (*P* > 0.05). At the same time, other series of letters also have significant differences (*P* < 0.05). **(A)** is the total Cr content of soil; **(B)** is the content of soil Cr^6+^; **(C)** is the total Cr content in plant leaves; **(D)** is the content of Cr^6+^ in plant leaves. CK0 (0 mg*kg^−1^ K_2_Cr_2_O_7_, Sampling on the 0th day), Cr7 (100 mg*kg^−1^ K_2_Cr_2_O_7_, Sampling on the 7th day), Cr14 (100 mg*kg^−1^ K_2_Cr_2_O_7_, Sampling on the 14th day), and Cr21 (100 mg*kg^−1^ K_2_Cr_2_O_7_, Sampling on the 21th day). CK7 (0mg*kg^−1^ K_2_Cr_2_O_7_, Sampling on the 7th day), CK14 (0mg*kg^−1^ K_2_Cr_2_O_7_, Sampling on the 14th day), and CK21 (0mg*kg^−1^ K_2_Cr_2_O_7_, Sampling on the 21th day).

When Cr (100 mg/kg) was added, the pH and EC of plant rhizosphere soil showed a gradually decreasing trend over time (Supplementary Figures S1E, F). At the same time, the soil organic matter (SOM) content was lower than that of the control group only in Cr7, and the maximum value was found in Cr14 (Supplementary Figure S1D). In addition, plant biomass significantly decreased after Cr stress (Supplementary Figure S1C). In addition, the content of Cr adsorbed by plants was measured in this study (Figures 1A–D). After adding Cr, the soil's content gradually decreased with the extension of stress time, while the content of Cr in plant leaves decreased significantly (Figures 1A, C). Meanwhile, the content of Cr(VI) in soil and plant leaves was significantly higher than that in the control group (Figures 1B, D).

**Figure 2:**
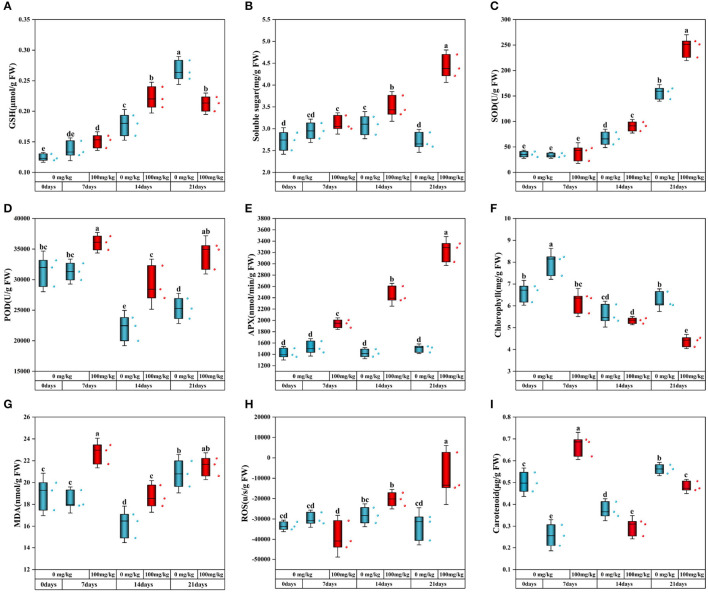
Physiological and biochemical indexes of *C. indica* [**(A)** GSH; **(B)** soluble sugar; **(C)** SOD; **(D)** POD; **(E)** APX; **(F)** chlorophyll; **(G)** MDA; **(H)** ROS; **(I)** carotenoid]. There was no significant difference in data within the same interval (*P* > 0.05). At the same time, other series of letters also have significant differences (*P* < 0.05). CK0 (0 mg*kg^−1^ K_2_Cr_2_O_7_, Sampling on the 0th day), Cr7 (100 mg*kg^−1^ K_2_Cr_2_O_7_, Sampling on the 7th day), Cr14 (100 mg*kg^−1^ K_2_Cr_2_O_7_, Sampling on the 14th day), and Cr21 (100 mg*kg^−1^ K_2_Cr_2_O_7_, Sampling on the 21th day). CK7 (0mg*kg^−1^ K_2_Cr_2_O_7_, Sampling on the 7th day), CK14 (0mg*kg^−1^ K_2_Cr_2_O_7_, Sampling on the 14th day), and CK21 (0mg*kg^−1^ K_2_Cr_2_O_7_, Sampling on the 21th day).

In this study, the content changes of antioxidant indexes and oxidative stress indexes were measured. Compared with no addition of Cr, the content of GSH in plants under Cr stress gradually increased with time but significantly decreased at 21 days (Figure 2A). The soluble sugar content increased significantly with the extension of stress time after Cr was added (Figure 2B). When Cr stressed plants, the oxidative stress process of resistance to stress caused changes in MDA and ROS levels (Figures 2G, H). Compared with the control group, MDA content in Cr-contaminated group increased significantly on days 7 and 14 but had no significant change on day 21. The ROS content of Cr-stressed plants increased gradually with the passage of stress time and reached its peak on day 21.

SOD activity of Cr-treated plants increased significantly with prolonged stress time, and the trend of SOD activity in the control group was similar (Figure 2C). Compared with the control group, POD and APX activities of Cr-treated plants were higher than those of the control group, with an average increase of 34.70 and 115.11% at 21 days, respectively (Figures 2D, E).

When Cr stressed plants, the content of photosynthetic pigment changed. Compared with the control group, chlorophyll content was lower after Cr addition and gradually decreased with the passage of the stress time sequence (Figure 2F). The contents of chlorophyll a and chlorophyll b increased with the increase of stress time (Supplementary Figures S1A, B). In addition, compared with the experimental group, only the carotenoid content in the Cr7 group increased significantly by 27.52%, and the other two groups were lower than the control group (Figure 2I).

### 3.2. Effects of Cr stress and rhizosphere effects on the distribution and diversity of soil bacterial communities

**Figure 3:**
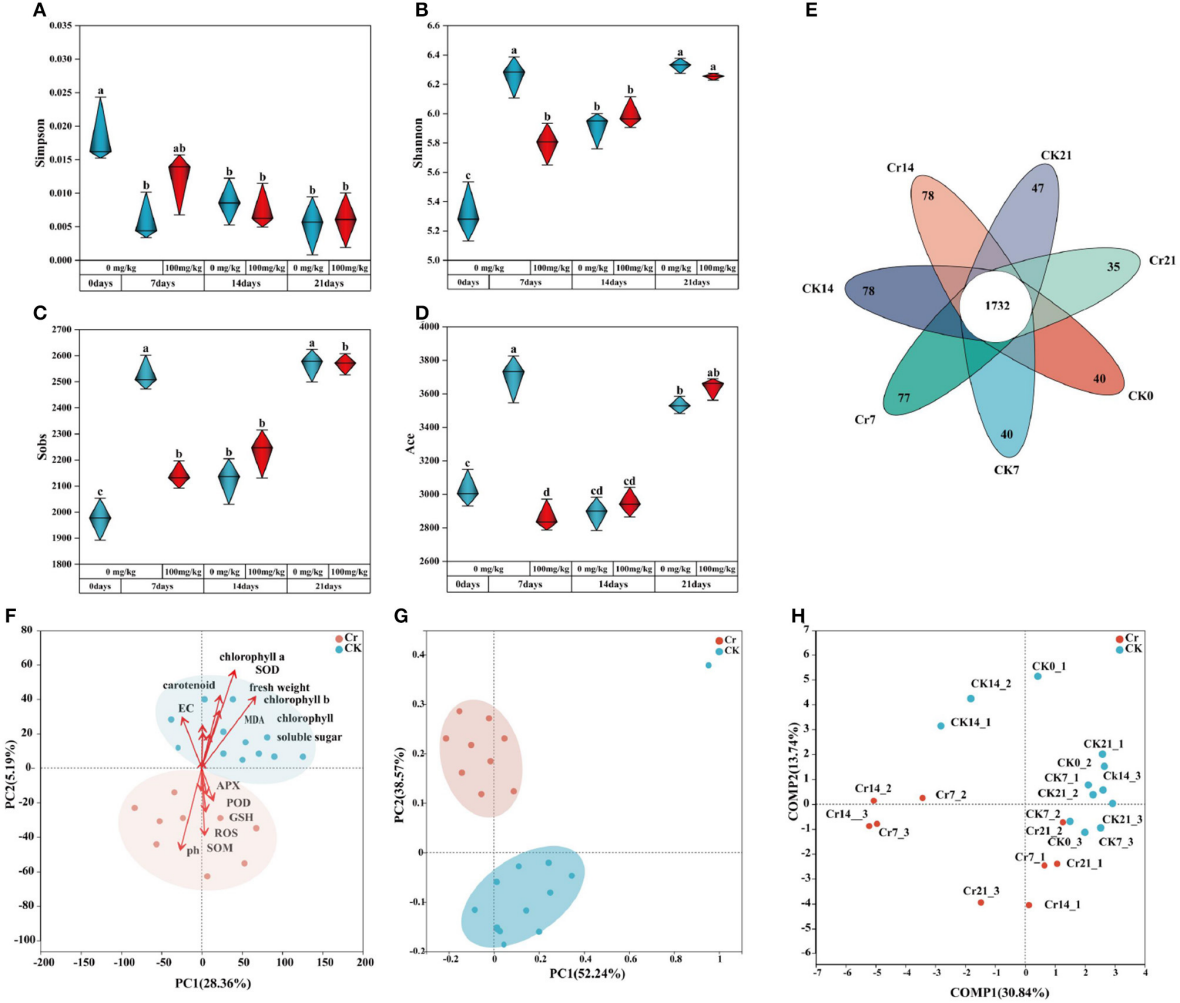
Analysis of rhizosphere soil microbial diversity and richness **(A–D)**. **(E)** is an Venn diagram of the rhizosphere microbial community. The overlapped part is shared OTU, and the non-overlapped part is unique OUT for each group. PCA analysis **(F)**, PCoA analysis **(G)**, and PLS-DA analysis **(H)** were performed for weighted UniFrac distance. CK0 (0 mg*kg^−1^ K_2_Cr_2_O_7_, Sampling on the 0th day), Cr7 (100 mg*kg^−1^ K_2_Cr_2_O_7_, Sampling on the 7th day), Cr14 (100 mg*kg^−1^ K_2_Cr_2_O_7_, Sampling on the 14th day), and Cr21 (100 mg*kg^−1^ K_2_Cr_2_O_7_, Sampling on the 21th day). CK7 (0mg*kg^−1^ K_2_Cr_2_O_7_, Sampling on the 7th day), CK14 (0mg*kg^−1^ K_2_Cr_2_O_7_, Sampling on the 14th day), and CK21 (0mg*kg^−1^ K_2_Cr_2_O_7_, Sampling on the 21th day).

To investigate the diversity of rhizosphere microbial community, α diversity analysis was performed. The sequencing quantity was sufficient according to the dilution curve (Supplementary Figure S2). As shown in Figures 3A–D, compared with the control group, *C. indica* of rhizosphere bacterial diversity index decreased and then increased after Cr contamination. On day 7, the bacterial diversity index in the Cr pollution group was significantly lower than that in the control group. After Cr was added, the timing changes resulted in the similarity and overlap of microbial composition (Figure 3E); 1,732 OTUs were shared between the groups, with each experimental and control group having a unique number of OTU (Figure 3E). Under Cr temporal stress, the Cr7 group produced more specific bacteria (77 OTUs) than the CK7 group (40 OTUs). The number of OTU in group Cr21 was 12 less than that in group CK21. In addition, there was no significant change in the number of OTUs between the Cr14 and CK14 groups.

β diversity analysis was conducted between the Cr treatment and CK groups. Principal component analysis (PCA) showed that the Cr treatment group and CK group were significantly partitioned, and environmental factors significantly induced the two zones, so the species composition of the experimental group and the control group was significantly different due to environmental factors (Figure 3F). Principal coordinate analysis (PCoA) was conducted for the experiment, and the figure showed apparent partitioning, which again verified that the species community composition was significantly different (Figure 3G). In addition, partial least squares discriminant analysis was carried out for experimental grouping arrangement, and the influence on both axes was uniform, that is, the experimental grouping arrangement was reasonable (Figure 3H).

### 3.3. Changes of rhizosphere microbial community structure and composition in *C. indica* under Cr stress

**Figure 4:**
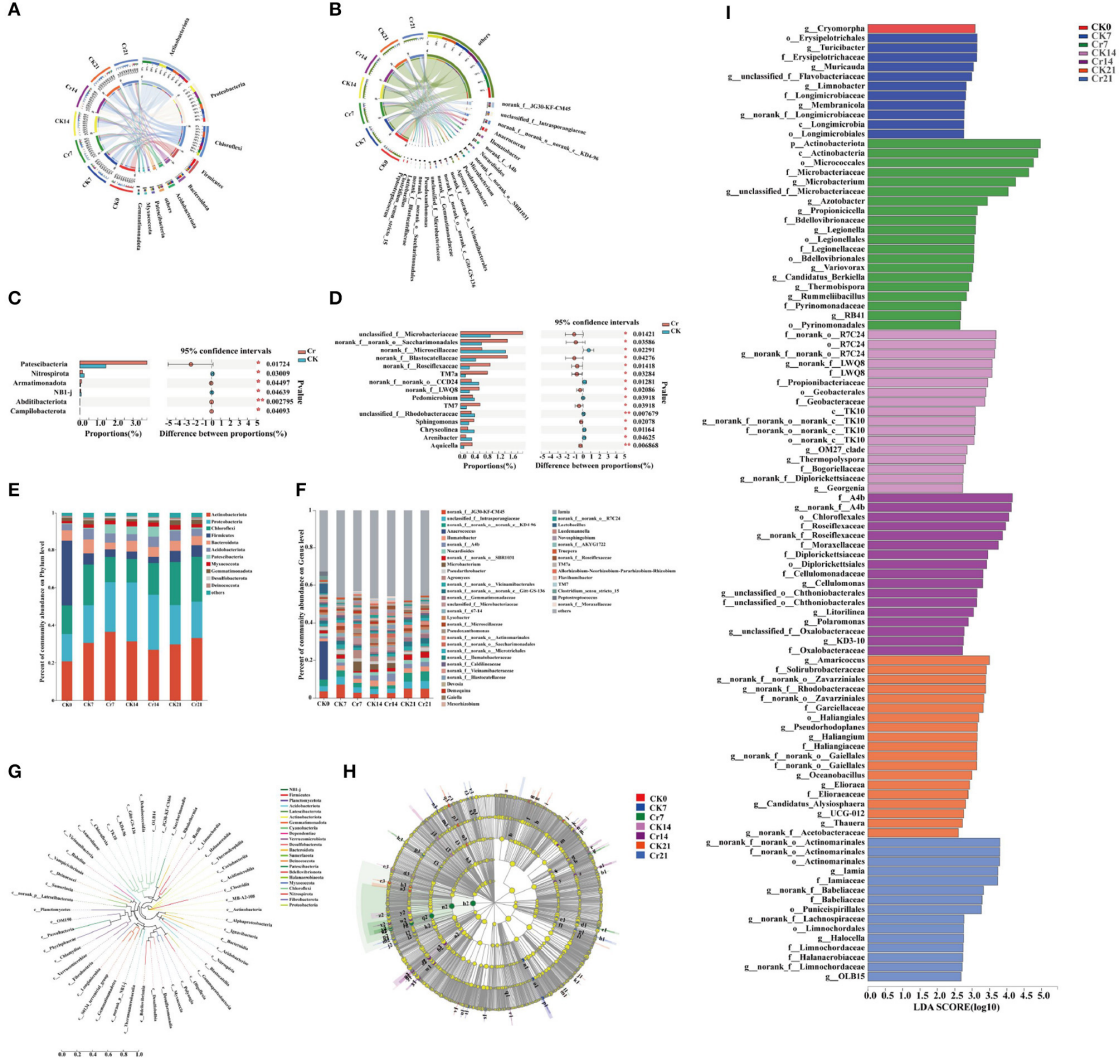
Microbial community structure and differences in rhizosphere soil of *C. indica* under different Cr stress time series [**(A)** phylum level; **(B)** genus level]. Significance test of differences between groups [**(C)** phylum level; **(D)** genus level]. Bar graph analysis of community composition [**(E)** phylum level; **(F)** genus level]. Each column of the species corresponds to the relative abundance of the species in each sample, with different colors representing different samples. In the confidence interval, the values corresponding to the points represent the differences in the relative abundance of the two samples. **(G)** A ring evolutionary tree constructed by the flora of different groups. Analysis of multilevel LEfSe species differences among different groups [**(H)** tree; **(I)** LDA discrimination histogram, the larger the LDA value, the greater the abundance difference; LDA scales > 3]. Cr0 (0 mg^*^kg^−1^ K_2_Cr_2_O_7_, Sampling on the 0th day), Cr7 (100 mg^*^kg^−1^ K_2_Cr_2_O_7_, Sampling on the 7th day), Cr14 (100 mg^*^kg^−1^ K_2_Cr_2_O_7_, Sampling on the 14th day), and Cr21 (100 mg^*^kg^−1^ K_2_Cr_2_O_7_, Sampling on the 21th day). CK7 (0 mg^*^kg^−1^ K_2_Cr_2_O_7_, Sampling on the 7th day), CK14 (0 mg^*^kg^−1^ K_2_Cr_2_O_7_, Sampling on the 14th day), and CK21 (0 mg^*^kg^−1^ K_2_Cr_2_O_7_, Sampling on the 21th day).

In this study, the rhizosphere microbial community of *C. indica* was analyzed. It turns out that at the phylum level, the bacteria in each group are *Actinobacteria, Proteobacteria, Chloroflexi, Firmicutes, Bacteroidota, Acidobacteriota, Patescibacteria, Myxococcota*, and *Gemmatimonadota* (Figure 4A). However, at the genus level, the bacteria in each group are *norank_f__JG30-KF-CM45, Anaerococcus*, and *Ilumatobacter* (Figure 4B). In the first 15 phyla of the Cr treatment group compared to the CK group, the bacteria with the most apparent abundance difference are *Patescibacteria, Nitrospirota, Armatimonadota, NB1-j, Abditibacteriota*, and *Campilobacterota* (Figure 4C). As shown in Figures 4E, F, the abundance of different dominant bacteria in each group was different. Under the phylum category, members of the dominant bacterial groups include *Actinobacteria* (20.71–36.72%), *Proteobacteria* (14.54–31.36%), *Chloroflexi* (12.40–23.77%), *Firmicutes* (3.27–34.42%), *Bacteroidota* (3.54–7.42%), *Acidobacteriota* (3.55–5.40%), *Patescibacteria* (0.49–5.43%), *Myxococcota* (1.30–2.85%), and *Gemmatimonadota* (1.08–2.75%). Under the genus classification, the clear distinction of dominant community highlighted the diversity of the community. At the phylum level, evolutionary relationships among species of plant root bacteria in each experimental group were analyzed, and a phylogenetic ring tree was constructed (Figure 4G). In addition, Cladograms (Figure 4H) and LDA (Figure 4I) analyzed by linear discriminant analysis effect size (LEfSe) were used in this study to reveal key biomarkers of different communities. The clade diagram showed that after adding Cr, one phylum, one order, and one genus of *Actinobacteria* in Group Cr7 were enriched. There is one class, two orders, one family, and one genus in Group Cr14. However, only one class and two genera were enriched in the Cr21 group. From the perspective of time sequence, the Cr7 group can detect more bacterial groups. At the generic level, in the Cr7 group, *Microbacterium, Azotobacter, Propionicicella, Legionella, Variovorax, Candidatus_Berkiella, Thermobispora, Rummeliibacillu*, and *RB41* were obtained. *Norank_f__A4b, Roseiflexaceae, Cellulomonas, unclassified_o__Chthoniobacterales, Litorilinea, Polaromonas, unclassified_f__Oxa lobacteraceae*, and *KD3-10* were enriched in Cr14 group. Furthermore, *norank_f__norank_o__Actinomarinales, Iamia, norank_f__Babeliaceae, norank_f__Lachnospiraceae, Halocella, norank_f__Limnochordaceae*, and *OLB15* were enriched in the Cr21 treatment group. To well explain the trend of dominant community among samples in the direction of race, an evolutionary tree analysis was constructed (Supplementary Figure S2), and according to the ratio of Reads, *Actinobacteria, Alphaproteobacteria, Gammaproteobacteria*, and *Acidimicrobiia* had significant changes between the control group and the polluted group after Cr stress treatment.

### 3.4. Correlation analysis between rhizosphere microbial community and physiological indexes of *C. indica*

**Figure 5:**
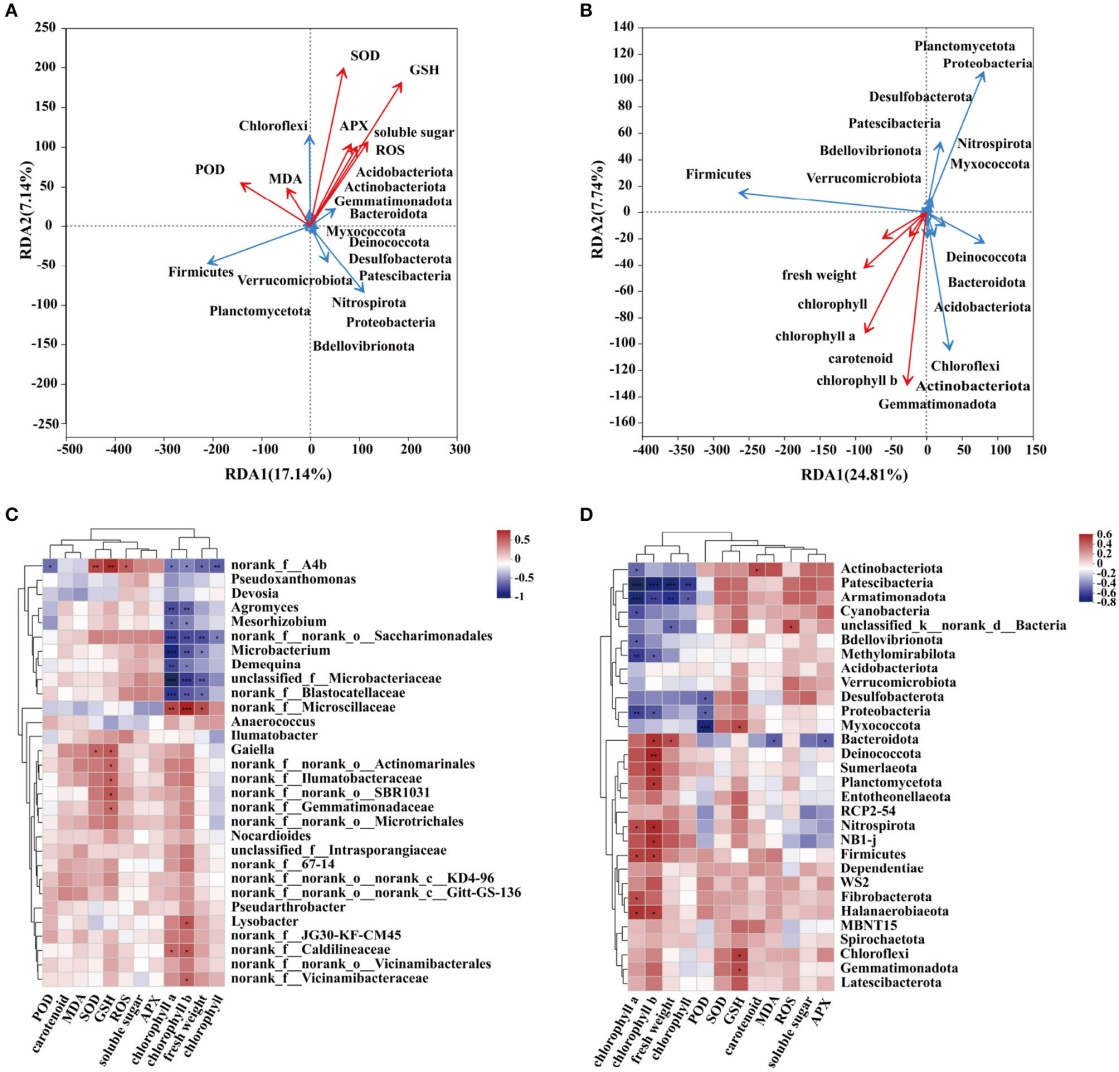
Correlation analysis between microflora and environmental factors. **(A)** The correlation between antioxidant reaction and dominant flora. **(B)** The correlation between plant physiological indexes and dominant flora. heatmap analysis of correlations between environmental factors and dominant flora [**(D)** phylum level, **(C)** genus level]. R-values are shown in different colors in the figure. If p-values are less than 0.05, they are marked by ^*^. The legend on the right is the color range of different R-values. You can choose to present a cluster tree of species and environmental factors (e.g., left and top). (^*^ is *P* ≤ 0.05, ^**^ is *P* ≤ 0.01, ^***^ is *P* ≤ 0.001).

To verify the correlation between physiological and biochemical indexes and the dominant community, redundancy analysis and correlation heat map analysis were conducted in this experiment (Supplementary Table S3). The redundancy analysis results showed that plants' oxidative stress and antioxidant indexes were correlated with some dominant microbial communities. The soluble sugar content of SOD, GSH, and APX is positively correlated with *Acidobacteriota, Actinobacteriota, Gemmatimonadota, Bacteroidota*, and *Chloroflexi*. In addition, these dominant bacteria showed the same trend as ROS and MDA contents. On the contrary, there is a negative correlation with POD (Figure 5A). Fresh weight and photosynthetic pigment content were positively correlated with *Deinococcota, Bacteroidota, Acidobacteriota, Chloroflexi, Actinobacteriota*, and *Gemmatimonadota*. They were negatively correlated with other dominant communities (Figure 5B). To show the significance of the correlation between physiological and biochemical indexes and the related community, a correlation heat map analysis was conducted (Figure 5D). Heat map analysis showed that chlorophyll a, b, and fresh weight were negatively correlated with *Patescibacteria* and *Armatimonadota*. Meanwhile, chlorophyll a was negatively correlated with *Methylomirabilota* and *Proteobacteria*. However, chlorophyll a and b were significantly positively correlated with *Nirmicutes, Firmicutes*, and *Halanaerobiaeota*. POD and *Myxococcota* showed a significant negative correlation. GSH was positively correlated with *Chloroflexi* and *Gemmatimonadota*. Figure 5C shows the significant analysis of physiological and biochemical indexes and bacterial communities at the genus level. Chlorophyll a, chlorophyll b, *norank_f__norank_o__Saccharimonadales, Microbacterium, Demequina, unclassified_f__Microbacteriaceae*, and *norank_f__Blastocatellaceae* were significantly negatively correlated, while *norank_f__Microscillaceae* and *norank_f__Caldilineaceae* were significantly positively correlated.

### 3.5. KEGG pathway enrichment analysis

**Figure 6:**
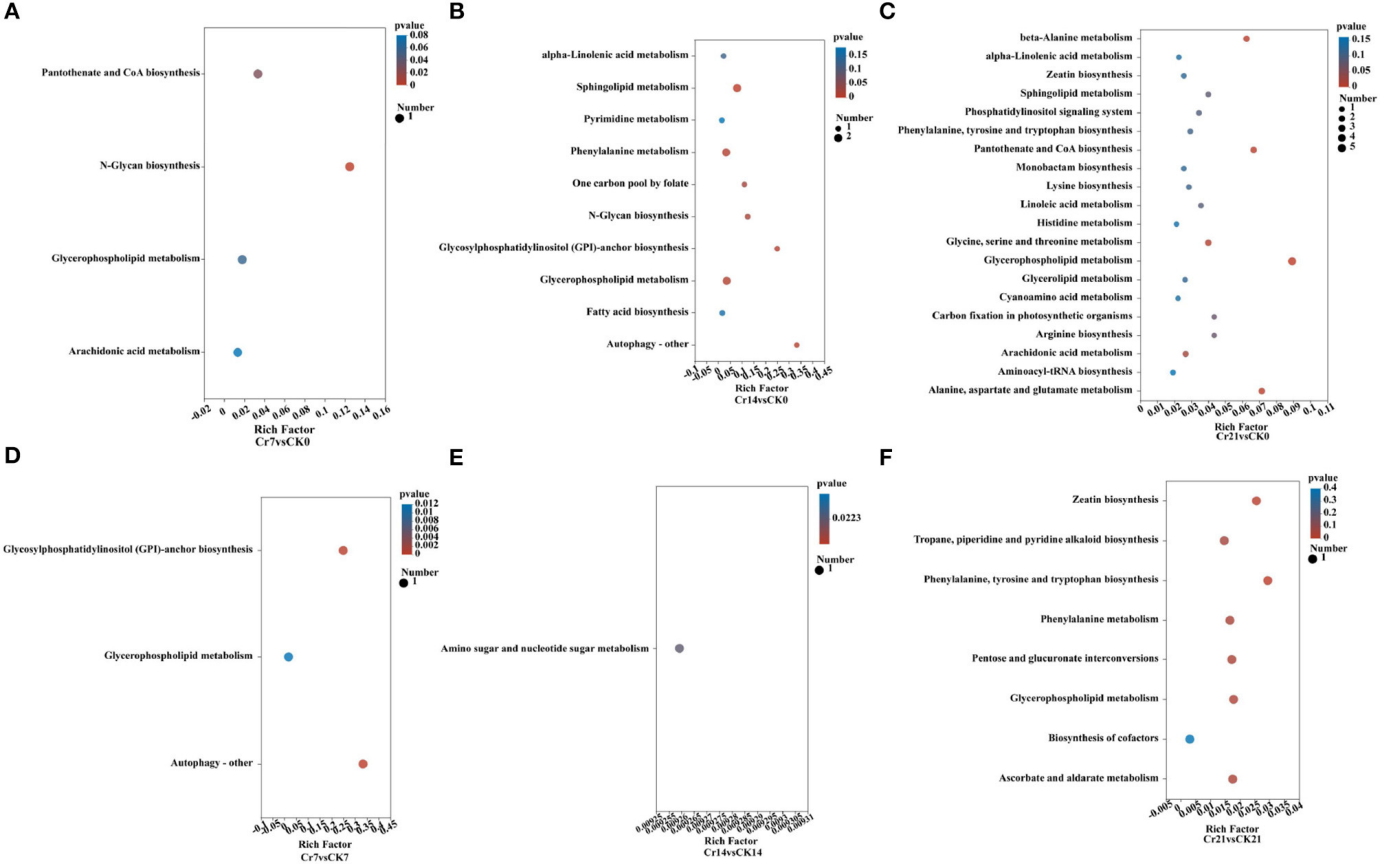
KEGG pathway enrichment analysis. The abscissa is enrichment rate, and the ordinate is KEGG collateralization [**(A)** Cr7vsCK7; **(B)** Cr14vsCr0; **(C)** Cr21vsCr0; **(D)** Cr7vsCK7; **(E)** Cr14vsCK14; **(F)** Cr21vsCK21]. The size of bubbles in the figure represents the concentration of metabolic compound in this pathway, and the color of bubbles represents the P-values of different enrichment significations. CK0 (0 mg*kg^−1^ K_2_Cr_2_O_7_, Sampling on the 0th day), Cr7 (100 mg*kg^−1^ K_2_Cr_2_O_7_, Sampling on the 7th day), Cr14 (100 mg*kg^−1^ K_2_Cr_2_O_7_, Sampling on the 14th day), and Cr21 (100 mg*kg^−1^ K_2_Cr_2_O_7_, Sampling on the 21th day). CK7 (0mg*kg^−1^ K_2_Cr_2_O_7_, Sampling on the 7th day), CK14 (0mg*kg^−1^ K_2_Cr_2_O_7_, Sampling on the 14th day), and CK21 (0mg*kg^−1^ K_2_Cr_2_O_7_, Sampling on the 21th day).

KEGG pathway analysis showed a significant difference in the rhizosphere metabolite quantity ratio between Cr and CK groups. Compared with the group, the number of metabolites and metabolic enrichment pathways in the Cr21 group was the largest. Beta-alanine metabolism; pantothenate and CoA biosynthesis; alanine, aspartate, and glutamate metabolism; and glycerophospholipid metabolism are the most abundant pathways of *C. indica* rhizosphere metabolites (Figure 6C). While the Cr14 group compared with the blank group, the number of metabolites concentration factors of the two most prominent synthesis pathways is Autophagy - other, Glycosylphosphatidylinositol (GPI) - anchor biosynthesis; in contrast, in the comparison between the Cr7 group and the blank group, only the enrichment factor of the number of metabolites in the synthesis pathway of N-Glycan biosynthesis reached the maximum value and reached a significant level (Figures 6A, B). At the same time, the difference in metabolites between the control group and the corresponding time sequence after Cr addition was analyzed (Figures 6D–F). The results showed that the number of metabolites in the Cr21 vs. CK21 group was the largest, and the most significant enrichment pathways were phenylalanine, tyrosine, tryptophan biosynthesis, and zeatin biosynthesis. On the contrary, the number of differential metabolites in the Cr14 vs. CK14 and Cr7 vs. CK7 groups was the lowest. In addition, only the Cr7 vs. CK7 group has significant enrichment approaches; practical enrichment approaches are autophagy-other and glycosylphosphatidylinositol (GPI)-anchor biosynthesis. Therefore, the differential amount and enrichment pathway of plant rhizosphere metabolites increased gradually with the passage of stress time after Cr addition.

### 3.6. Effect of Cr stress on differential expression of metabolites in roots of *C. indica*

**Figure 7:**
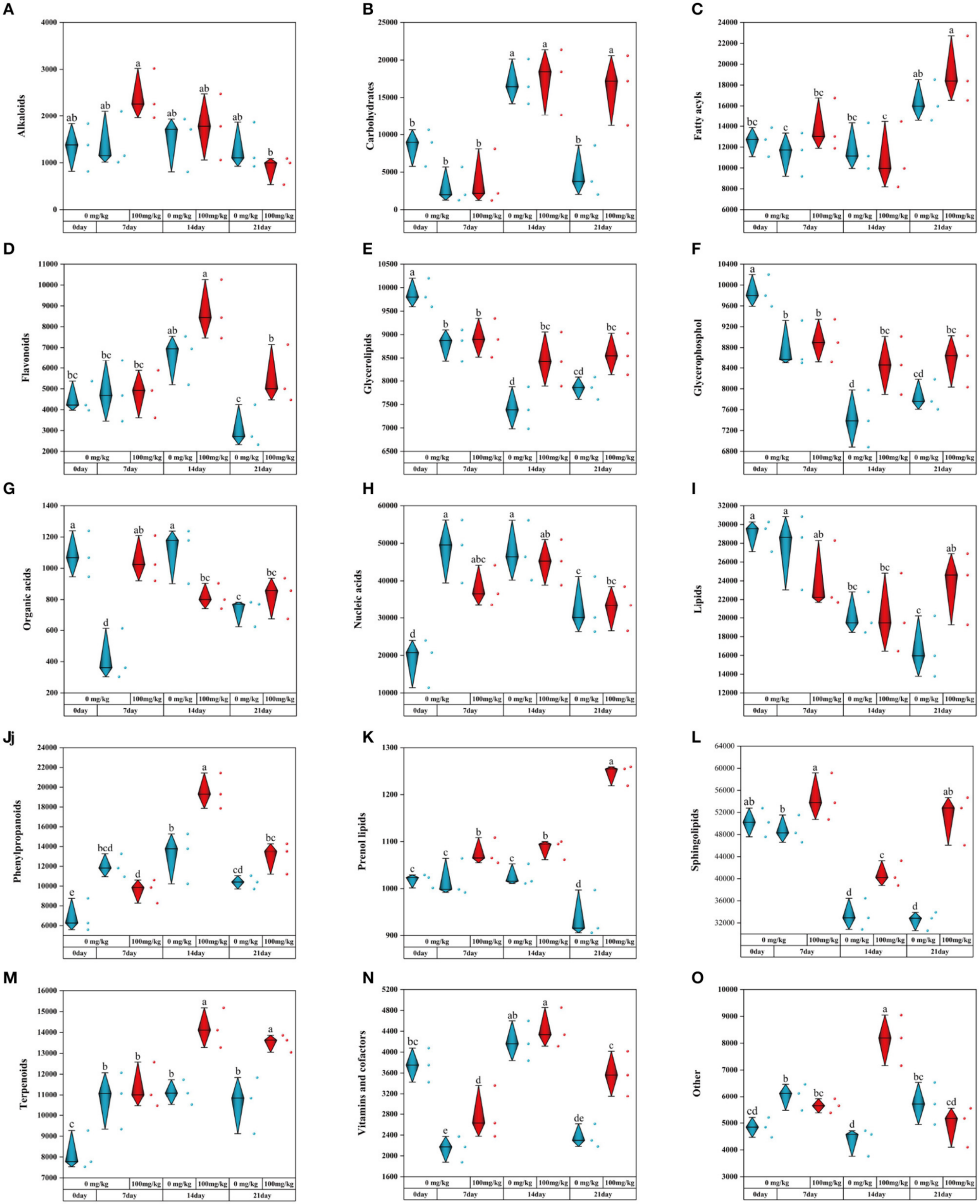
The contents of plant metabolites under Cr treatment [**(A)** Alkaloids; **(B)** Carbohydrates; **(C)** Fatty acyls; **(D)** Flavonoids; **(E)** Glycerolipids; **(F)** Glycerophosphol; **(G)** Organic acids; **(H)** Nucleic acids; **(I)** lipids; **(J)** Phenylpropanoids; **(K)** Prenol lipids; **(L)** Sphingolipids; **(M)** Terpenoids; **(N)** Vitamins; and cofactors; **(O)** Other]. There was no significant difference in data within the same interval (*P* > 0.05). At the same time, other series of letters also have significant differences (*P* < 0.05). CK0 (0 mg*kg^−1^ K_2_Cr_2_O_7_, Sampling on the 0th day), Cr7 (100 mg*kg^−1^ K_2_Cr_2_O_7_, Sampling on the 7th day), Cr14 (100 mg*kg^−1^ K_2_Cr_2_O_7_, Sampling on the 14th day), and Cr21 (100 mg*kg^−1^ K_2_Cr_2_O_7_, Sampling on the 21th day). CK7 (0mg*kg^−1^ K_2_Cr_2_O_7_, Sampling on the 7th day), CK14 (0mg*kg^−1^ K_2_Cr_2_O_7_, Sampling on the 14th day), and CK21 (0mg*kg^−1^ K_2_Cr_2_O_7_, Sampling on the 21th day).

To explore the changes of metabolic compounds in the rhizosphere of *C. indica* after adding Cr, a statistical analysis of metabolic compounds with high content was conducted by SPSS (27.0). The results showed that 14 metabolites changed significantly with Cr stress time. Compared with a control group, the contents of alkaloids Cr processing significantly promoted, but will gradually decrease with the stress on long (Figure 7A). In the meanwhile, the content of carbohydrates in the Cr21 and CK21 groups was 241.14% higher on average than carbohydrates in the other two groups (Figure 7B). It is worth noting that in the early stage of Cr stress, the content of fatty acyls did not change significantly compared with that of CK0 group, but increased significantly at day 21 (Figure 7C). Flavonoids in Cr treatment group at days 14 and 21 are 32.94 and 79.06% more than CK treatment group, respectively (Figure 7D). At the same time, the contents of glycerolipids and glycerophosphol in the Cr treatment group were higher than those in the control group, while the contents of organic and nucleic acids had no significant change (Figures 7E–H). In Figure 7I, lipid content changed significantly only on the 21st day, 41.69% more in the experimental group than in the control group. Under Cr treatment, the content of phenylpropanoids increased first and then decreased with the duration of stress, while the content of sphingolipids showed the opposite trend (Figures 7J, L). In addition, the content of the prenol lipids treatment group was significantly higher than that of the control group, with the maximum peak at day 21 (Figure 7K). After the addition of Cr, terpenoids increased by 27.67 and 27.53% at 14 and 21 days (Figure 7M). There were also changes in vitamins and cofactors in the Cr treatment group, over time of Cr stress. However, the metabolite content in the 14 treatment groups was the highest in the other groups (Figures 7N, O). Rhizosphere metabolic changes can improve the rhizosphere bacteria groups and the rhizosphere environment of plant growth to improve the survival ability of plants in Cr pollution.

## 4. Discussions

### 4.1. Mechanism of rhizosphere bacteria and *C. indica* response to Cr stress

As we all know, high or low heavy metal concentrations harm plants. Excessive heavy metals in plants will destroy cell structure, affect photosynthesis, and cause an imbalance of redox homeostasis (Yaashikaa et al., 2022). To reduce the toxicity of heavy metals, plants secrete antioxidants and recruit rhizosphere microorganisms to ensure the growth and development of plant bodies and high adaptability to the environment. In this study, MDA and ROS contents showed an increasing trend with the extension of Cr stress time (Figures 2G, H). To cope with the damage of free radicals in plants under Cr stress, *C. indica* would synthesize vitamin C, vitamin E, carotenoids, polyphenols, and other substances to protect cells from oxidative stress (Zhang et al., 2013). This may be why the high carotenoid content in Cr stress groups is under different time sequences (Figure 2I). The rhizosphere microorganisms of *C. indica* also provide a sure guarantee for plant nutrition acquisition and root growth. Under Cr stress, rhizosphere microorganisms will secrete organic acids and extracellular polysaccharides to promote the complex precipitation of heavy metal ions in the soil, thus reducing plants' absorption of heavy metals (Hasanuzzaman et al., 2018). At the same time, rhizosphere microorganisms synthesized thalli and secreted organic acids to promote plant detoxification of heavy metals (Xia et al., 2021). In this study, the abundance and diversity of root microbial communities changed under Cr stress (Figures 3A–D). Among them, the main dominant bacteria in the rhizosphere of *C. indica* are *Actinobacteria, Proteobacteria*, and *Chloroflexi* (Figure 4A). *Actinobacteria* exist in the rhizosphere of plants under biological and abiotic stress and can promote plant growth and reduce the harm of environmental stress (Jiang et al., 2020; Ganesh et al., 2022). The weakening and elimination of abiotic stress by rhizosphere microorganisms may be due to the methylation modification of DNA in roots induced by plant growth-promoting bacteria (PGPB), thus alleviating the toxic effect of stress on the environment (Chen et al., 2022). Under the stress of Cd, the addition of nano-selenium can improve rhizosphere bacteria (e.g., *Gammaproteobacteria, Alphaproteobacteria, Bacteroidia, Gemmatimonadetes, Deltaproteobacteria*, and *Anaerolineae*) showed increased abundance to varying degrees (Li et al., 2022). Previous studies have found that the increase in the abundance of *Acidobacteriota* can promote the absorption of Cd by plants because organic acids secreted by *Acidobacterium* can promote the release of Cd from soil colloids (Ganesh et al., 2022). In this study, with the passage of Cr stress time, the abundance of *Acidobacteriota* decreased to 3.82% in Group Cr21 (Figure 4E), which may be caused by the decrease of Cr content in the rhizosphere with the extension of Cr stress time (Wei et al., 2023c). Rhizosphere microorganisms can induce the production of flavonoid antioxidant substances in plants, thereby enhancing the resilience of plants to oxidative stress (Wang et al., 2022b). This explains the significant increase of flavonoids under Cr treatment in sequences at 14 and 21 days (Figure 7D). Rhizosphere bacteria also produce antioxidant enzymes in plants. In this study, the contents of SOD, POD, APX, and GSH in *C. medica* in the Cr treatment group were significantly higher than those in the CK group (Figures 2A, C, D, E), which could be attributed to the antioxidant stress of plants and the induction of rhizosphere microorganisms. Apart from protecting plants in the above ways, rhizosphere microorganisms can also induce plants to enhance their immunity, thus reducing the persecution of heavy metals. For example, under Cd stress, rhizosphere rhizobium *Burkholderia* sp. *D54* can increase the activity of antioxidant enzymes in tomatoes and also activate PR proteins and transcription factors in plants to improve the immunity of wheat and reduce the damage of wheat under Cd stress (Mondal et al., 2021). In conclusion, in addition to responding well to Cr stress in *C. indica* plant body, rhizosphere microorganisms also significantly enhance heavy metal tolerance and the growth and development of plants under Cr stress.

### 4.2. Recruitment of microorganisms from rhizosphere secretions of *C. indica* under Cr stress

Rhizosphere-dominant bacteria and plant roots can form a dynamic rhizosphere environment. On the one hand, dominant bacteria may promote the synthesis of root exudates; on the other hand, root exudates may also provide energy for dominant bacteria (Sasse et al., 2018). For example, adding nitrogen changes the connections between plants and microbes (Liu and Zhang, 2019). In addition, *Arabidopsis thaliana* has different rhizosphere microbial communities due to its different development stages (seedling stage, vegetative stage, bolting stage, and flowering stage), and root exudates are positively correlated with certain bacterial genera such as *Acidobacteria, Actinomyces*, and *Bacteroidetes* (Chaparro et al., 2014; Herms et al., 2022). The metabolic activities of roots produce components of root exudates, and the compounds in root exudates may also be products of root metabolic processes. They are involved in the interaction and regulation of root systems and soil environment (Huang et al., 2019). This study showed that root exudates were divided into 15 types, including lipids, alkaloids, flavonoids, lipoyl groups, and terpenoids, after adding Cr. These metabolites may recruit specific bacterial community (Figure 7). The specific cucurbitacin in bitter triterpenoids selectively enriched *Enterobacter* and *Bacillus* in melon roots to form rhizosphere bacteria (Zhong et al., 2022). A review by Santoyo (2022) showed that legumes that secrete phenolic compounds, such as flavonoids and isoflavones, induce gene expression in rhizobia, resulting in the mutually beneficial symbiosis between rhizobia and legumes. In addition, Fang et al.'s (2021) research on the relationship between rhizosphere microorganisms and root secretions of three species of aquatic plants showed that the concentration of organic carbon secreted by the root system of *Phragmites australis* was higher than that of *Typha angustifolia L*. and *Cyperus alternifolius L*. Thus, *Cytohagaceae* had the highest abundance. At the same time, there was a significant consistency between the rhizosphere bacterial community and metabolites at different concentrations of high and low Cd concentrations in *Brassica napus L*. (Wu et al., 2023). Based on the analysis of the close relationship between root exudes and bacterial community differences under Cr stress under different time sequences, it is speculated that the differences in *C. indica* rhizosphere exude determine the abundance change and composition of the rhizosphere microbial community.

### 4.3. Key metabolic stress response mechanisms of *C. indica* under Cr stress

**Figure 8:**
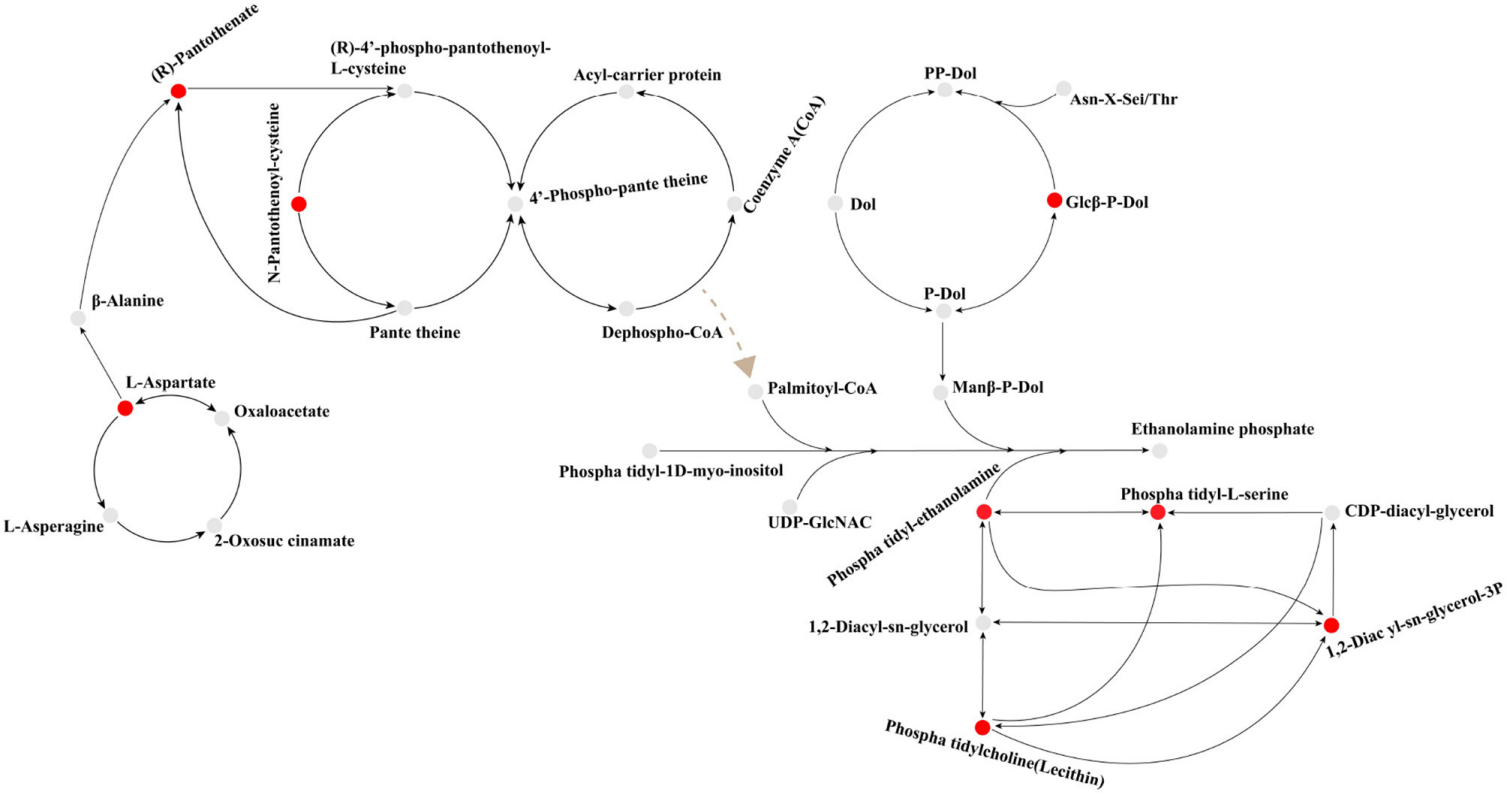
Key Biosynthetic pathways of rhizosphere metabolites in Canna canna under Chromium stress. Based on the data generated by the KEGG database and with some modifications, the path was determined. Red balls indicate up-regulation, while gray balls indicate no significant change. The black arrow shows the synthesis pathway. The dotted brown arrow indicates that it promotes the formation of a certain metabolite, but the synthetic pathway is unknown.

Plants release some organic substances, such as carbohydrates, amino acids, and organic acids, through root secretions, which can provide nutrients and energy sources for inter-root microorganisms. At the same time, inter-root microorganisms can also provide nutrients to plants by decomposing organic substances in the soil and fixing nitrogen. In addition, inter-root microorganisms can interact with plants to promote plant growth and health, for example, by inhibiting the growth of plant pathogens or increasing the plant's ability to absorb nutrients (Hu et al., 2018; Rodriguez et al., 2019; Wang et al., 2022a). Thus, a complex and mutually beneficial symbiotic system is formed between plants, root secretions and inter-root microorganisms. In this study, the inter-root metabolic secretions of *C. indica* under Cr stress were dominated by lipids, alkaloids, flavonoids, terpenoids, and organic acids (Figure 7). It mainly contains five critical metabolic pathways [glycerophospholipid metabolism; N-glycan biosynthesis; glycosylphosphatidylinositol (GPI)-anchor biosynthesis; pantothenate and CoA biosynthesis; alanine, aspartate, and glutamate metabolism; Figure 8]. There is a close connection among them; the differentially expressed metabolites involved are 79 (Supplementary Table S4). Root secretions act as a bridge and catalyst between plants and inter-rooted microorganisms. Also, root secretions can influence the community structure and function of inter-rooted microorganisms by changing the chemical and biological properties of the soil environment, thus regulating the stability and health status of the soil ecosystem (Zhalnina et al., 2018). Among the root, secretions are partly produced by secondary metabolisms, such as alkaloids, flavonoids, terpenoids, phenylpropanoids, phenolic acids, and saponins. These compounds have different roles and functions for plant growth and development, environmental adaptation, defense against diseases, attraction of pollinators, etc. (Pang et al., 2021). This is the reason for the increased content of flavonoids, terpenoid substances, and alkaloids in this study (Figure 7). In addition, the lipid metabolite synthesis pathway was significantly upregulated in this study, that is, the glycosylphosphatidylinositol (GPI)-anchor biosynthesis (Figure 6B), thus promoting a significant increase in lipid content. Most lipid metabolites, myristic acid, and 3-O-sulfolactose ceramide act as energy storage, biofilm skeleton stabilizer, and antioxidant after plant stress (Ma et al., 2021). Meanwhile, the carbohydrate content of plants in all Cr-treated groups in this study was higher than that of the control group (Figure 7B) because carbohydrates, an important class of organic substances, have a variety of biological activities and functions. Carbohydrates provide energy for inter-root microorganisms to survive and change the suitable soil structure for microorganisms, thus promoting plant growth (Ding et al., 2019). Microorganisms colonize roots due to the chemical induction and signal transduction of root secretions and the attachment on the root surface. In turn, inter-root microbes sense signals secreted by plant roots and release various signaling molecules to influence their hosts, enhance biotic and abiotic resistance, and promote root development and growth (Zhang et al., 2017).

## 5. Conclusion

This study revealed the correlation between *C. indica*, root metabolites, and rhizosphere bacteria under Cr stress. After the addition of Cr (100 mg^*^kg^−1^), the imbalance of oxidation balance and lipid peroxidation of plants increased ROS and MDA contents, which damaged the plant defense mechanism. To this end, plants' SOD, POD, and APX activities were increased. At the same time, the increase of GSH and soluble sugar content also had a positive effect on reducing Cr toxicity. In addition, the rhizosphere microbial diversity index of *C. indica* decreased after Cr addition, while the colonization abundance of dominant bacteria increased. There are 79 kinds of differentially expressed metabolites in the rhizosphere and five key metabolite generation pathways. The main categories of metabolites are terpenoids, lipids, flavonoids, and nucleic acids. In conclusion, by analyzing the antioxidant reaction of *C. indica* under Cr stress and the relationship between root secretion and bacterial community, this study provides a theoretical reference for plant antioxidant reaction and plant-bacterial interaction under heavy metal pollution. It provides new insights for heavy metal remediation.

## Data availability statement

The datasets presented in this study can be found in online repositories. The names of the repository/repositories and accession number(s) can be found in the article/Supplementary material.

## Author contributions

HM: writing—original draft. XY: investigation. LS: methodology. WZ: software. SZ: writing—review and editing. All authors contributed to the article and approved the submitted version.
