# Supplementary material for: Recruitment and metabolomics between Canna indica and rhizosphere bacteria under Cr stress
Source: Front Microbiol. 2023 Aug 15;14:1187982. doi: 10.3389/fmicb.2023.1187982 (PMC10465350; doi:10.3389/fmicb.2023.1187982)
Supplement: Supplementary file 1 [file Data_Sheet_1.pdf]

Fig.S1 Effects of Cr stress on soil physicochemical properties (e, pH;f, EC; d, SOM) and physiological and biochemical indices (c, Freshweight; a, chlorophyll a; b, chlorophyll b) of *C. indica*. There was no significant difference in data within the same interval ($P > 0.05$). At the same time, other series of letters also have significant differences ($P < 0.05$). Cr 0(0 mg*kg$^{-1}$ $K_2Cr_2O_7$, Sampling on the 0th day), Cr 7(100 mg*kg$^{-1}$ $K_2Cr_2O_7$, Sampling on the 7th day), Cr 14(100 mg*kg$^{-1}$ $K_2Cr_2O_7$, Sampling on the 14th day), and Cr 21(100 mg*kg$^{-1}$ $K_2Cr_2O_7$, Sampling on the 21th day). CK7 （0mg*kg$^{-1}$ $K_2Cr_2O_7$, Sampling on the 7th day)，CK14 （0mg*kg$^{-1}$ $K_2Cr_2O_7$, Sampling on the 14th day)，CK21 （0mg*kg$^{-1}$ $K_2Cr_2O_7$, Sampling on the 21th day).

Fig.S2 Bacterial community dilution curve of soil sample. The Alpha diversity index was used as the vertical coordinate to draw a curve, and the sufficient amount of sequencing data was judged according to whether the curve was flat. Cr0 was the blank group of the experiment. CK7, CK14 and CK21 respectively refer to the control group of the 7th, 14th and 21st days of the experiment. Cr7, Cr14, and Cr21 refer to the treatment group of the 7th, 14th, and 21st days of the experiment. Cr 0(0 mg*kg$^{-1}$ $K_2Cr_2O_7$, Sampling on the 0th day), Cr 7(100 mg*kg$^{-1}$ $K_2Cr_2O_7$, Sampling on the 7th day), Cr 14(100 mg*kg$^{-1}$ $K_2Cr_2O_7$, Sampling on the 14th day), and Cr 21(100 mg*kg$^{-1}$ $K_2Cr_2O_7$, Sampling on the 21th day). CK7 （0mg*kg$^{-1}$ $K_2Cr_2O_7$, Sampling on the 7th day)，CK14 （0mg*kg$^{-1}$ $K_2Cr_2O_7$, Sampling on the 14th day)，CK21 （0mg*kg$^{-1}$ $K_2Cr_2O_7$, Sampling on the 21th day).

Fig.S3 Evolution tree of rhizosphere microbial community of I. tectorum. On the left is the phylogenetic evolutionary tree. Each branch represents a species, and the branches are colored according to the higher taxonomic level to which the species belongs. The length of the branches is the evolutionary distance between the two species, that is, the degree of species difference. The bar chart on the right shows the proportion of Reads in different groups. Cr 0(0 mg*kg$^{-1}$ $K_2Cr_2O_7$, Sampling on the 0th day), Cr 7(100 mg*kg$^{-1}$ $K_2Cr_2O_7$, Sampling on the 7th day), Cr 14(100 mg*kg$^{-1}$ $K_2Cr_2O_7$, Sampling on the 14th day), and Cr 21(100 mg*kg$^{-1}$ $K_2Cr_2O_7$, Sampling on the 21th day). CK7 （0mg*kg$^{-1}$ $K_2Cr_2O_7$, Sampling on the 7th day)，CK14 （0mg*kg$^{-1}$ $K_2Cr_2O_7$, Sampling on the 14th day)，CK21 （0mg*kg$^{-1}$ $K_2Cr_2O_7$, Sampling on the 21th day).

Fig.S1

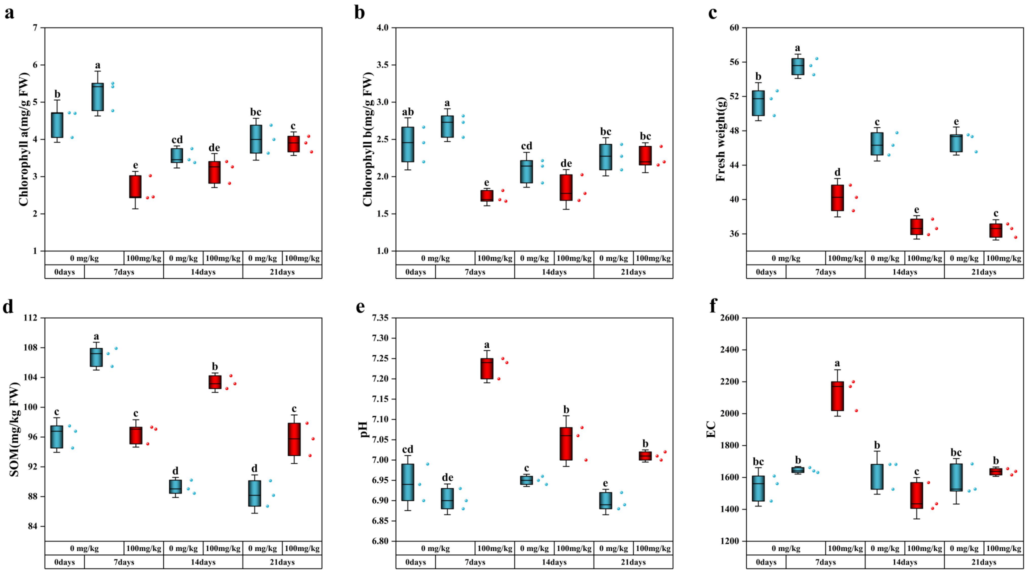
a
Chlorophyll a(mg/g FW)
b
Chlorophyll b(mg/g FW)
c
Fresh weight(g)
d
SOM(mg/kg FW)
e
pH
f
EC
0 mg/kg
100mg/kg
0days
7days
14days
21days

Fig.S2

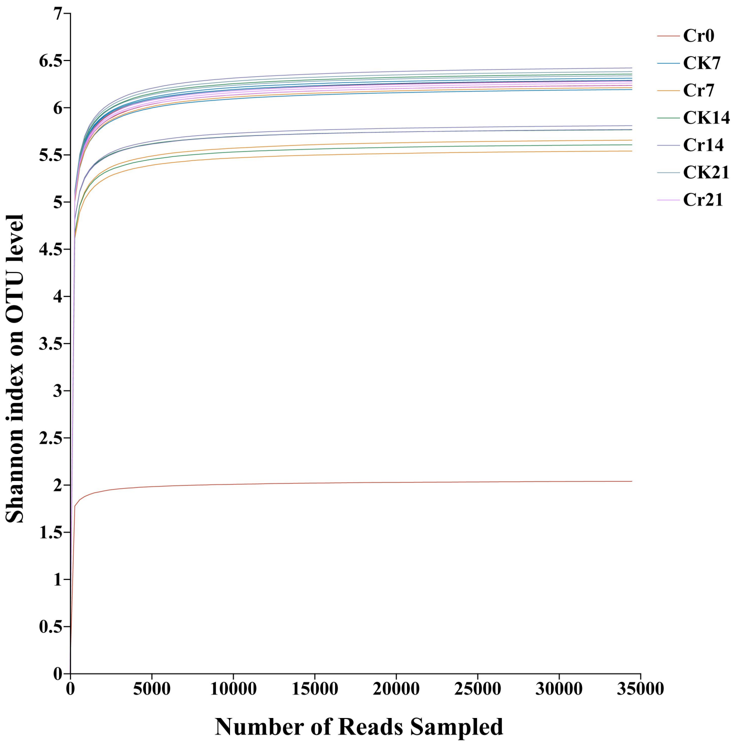

Shannon index on OTU level
Number of Reads Sampled
0
0.5
1
1.5
2
2.5
3
3.5
4
4.5
5
5.5
6
6.5
7
0
5000
10000
15000
20000
25000
30000
35000
Cr0
CK7
Cr7
CK14
Cr14
CK21
Cr21

Fig.S3

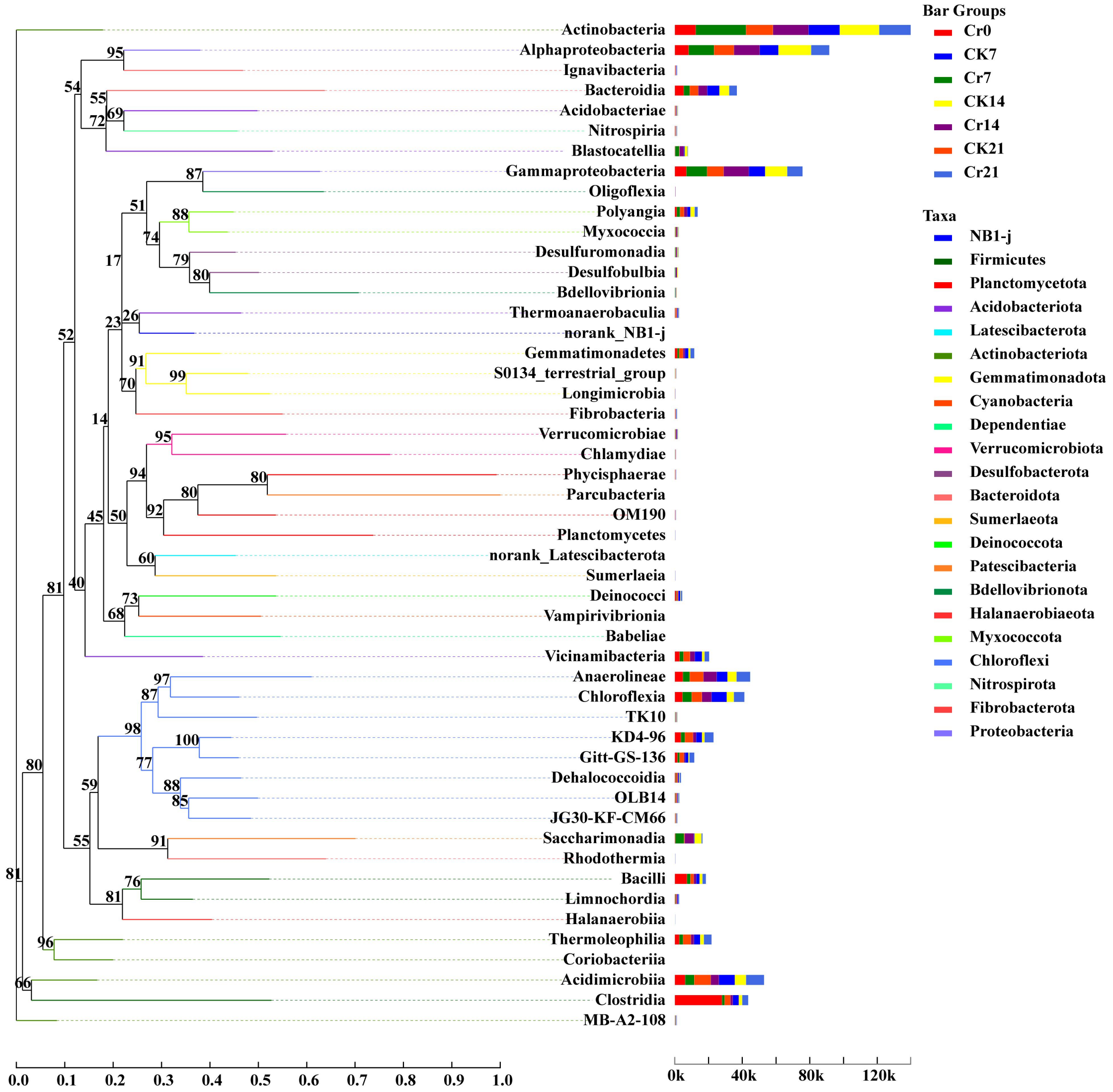

Bar Groups
Cr0
CK7
Cr7
CK14
Cr14
CK21
Cr21
Taxa
NB1-j
Firmicutes
Planctomycetota
Acidobacteriota
Latescibacterota
Actinobacteriota
Gemmatimonadota
Cyanobacteria
Dependentiae
Verrucomicrobiota
Desulfobacterota
Bacteroidota
Sumerlaeota
Deinococcota
Patescibacteria
Bdellovibrionota
Halanaerobiaeota
Myxococcota
Chloroflexi
Nitrospirota
Fibrobacterota
Proteobacteria
Actinobacteria
Alphaproteobacteria
Ignavibacteria
Bacteroidia
Acidobacteriae
Nitrospiria
Blastocatellia
Gammaproteobacteria
Oligoflexia
Polyangia
Myxococcia
Desulfuromonadia
Desulfobulbia
Bdellovibrionia
Thermoanaerobaculia
norank_NB1-j
Gemmatimonadetes
S0134_terrestrial_group
Longimicrobia
Fibrobacteria
Verrucomicrobiae
Chlamydiae
Phycisphaerae
Parcubacteria
OM190
Planctomycetes
norank_Latescibacterota
Sumerlaeia
Deinococci
Vampirivibrionia
Babeliae
Vicinamibacteria
Anaerolineae
Chloroflexia
TK10
KD4-96
Gitt-GS-136
Dehalococcoidia
OLB14
JG30-KF-CM66
Saccharimonadia
Rhodothermia
Bacilli
Limnochordia
Halanaerobiia
Thermoleophilia
Coriobacteriia
Acidimicrobiia
Clostridia
MB-A2-108
0.0 0.1 0.2 0.3 0.4 0.5 0.6 0.7 0.8 0.9 1.0
0k 40k 80k 120k
